# A randomised controlled trial of a faith-based culturally adapted intervention for depression in young Muslim women (IM-Adapted): a multi-site feasibility trial protocol

**DOI:** 10.1186/s40814-025-01691-9

**Published:** 2025-08-22

**Authors:** Megan Smith, Andy Jones, Pashtana Zormati, Louca-Mai Brady, Allan Clark, Atiya Kamal, Farzana Karawalli, Safiya Khan, Silvana E. Mengoni, Ghazala Mir, David Turner, Salman Waqar, David Wellsted, Daksha Trivedi

**Affiliations:** 1 Centre for Health Services and Clinical Research, University of Hertfordshire, Hatfield, AL10 9AB UK; 2 Centre for Research in Public Health and Community Care, School of Health and Social Work, University of Hertfordshire, Hatfield, AL10 9AB UK; 3 Norwich Medical School, University of East Anglia (UEA), Norwich, NR4 7TJ UK; 4 College of Psychology, Birmingham City University, 4 Cardigan Street, Birmingham, B4 7BD UK; 5 University of Hertfordshire, Hatfield, AL10 9AB UK; 6 Inspirited Minds, 175 Boundary Road, London, E17 8NG UK; 7 Leeds Institute of Health Sciences, University of Leeds, Leeds, LS2 9LN UK; 8 Department of Primary Care and Public Health, Imperial College London, London, W12 0BZ UK

**Keywords:** Depression, Low mood, Muslim, Women, Young people, Young adult, Cognitive behavioural therapy, Feasibility trial

## Abstract

**Background:**

Growing evidence suggests that mental health issues heavily impact Muslims, the largest, fastest growing minority religious group in the UK. High prevalence of anxiety and depression has been reported in young women aged 18 to 30 largely from Bangladeshi and Pakistani Muslim communities in Britain. Evidence suggests that incorporating religious and culturally adapted interventions may improve their effectiveness for Muslim populations, but their feasibility for young women in UK contexts is uncertain. This study aims to test whether a randomised controlled trial (RCT) of a faith-based intervention for young Muslim women living with depression is feasible.

**Methods:**

This study is a two-arm cluster randomised controlled feasibility trial with embedded process evaluation. Participants will be young Muslim women aged 18 to 24 years experiencing mild to moderate low mood or depression in Birmingham and London. The intervention will be delivered by trained therapists, supported by mental health support workers, once a week for 6 weeks. The two arms will be the IM-Adapted faith-based intervention and the standard NHS psychoeducation support group control with a proposed sample size of 30 per arm. Outcomes are referral, recruitment and retention rates, session attendance, adherence and acceptability of intervention, data collection, and adverse events, measured at baseline, 3 and 6 months.

**Discussion:**

The findings will provide early indication as to whether tailored mental health interventions may increase accessibility and effectiveness of support in underserved communities, addressing barriers linked to social and cultural factors. This will guide health services on the incorporation of cultural and religious adaptations in their programmes to better engage minority groups and improve mental health outcomes.

**Trial registration:**

ISRCTN, https://www.isrctn.com/ISRCTN17842222, registered 17th April 2024.

## Introduction

### Background and rationale

There is growing concern about the mental health and well-being of children and young people, with a striking sixfold increase in the prevalence of long-term mental health conditions in England from 1995 to 2014 in those aged under 24 [1]. Young people are particularly susceptible to the onset of mental illness, with over 50% of mental health problems emerging before the age of 14 and 75% before the age of 25 [2]. It is of concern that young women aged 16 to 24 have three times higher prevalence than men (26% vs 9%) with problems more common in young people from minority ethnic backgrounds [3, 4]. Females aged 24 years and under have seen the largest increase in suicide rates over the last three decades [5]. In England and Wales, rates amongst females aged 10 to 24 increased by 24% between 1992 and 2022, with a more substantial increase of around 41% over the last 20 years [5].

Growing evidence suggests that mental health issues can heavily impact Muslims; the largest, fastest growing minority religious group in the UK [6–9]. Particularly, high prevalence of anxiety and depression (over 35%) has been reported in young women aged 18 to 30 largely from Bangladeshi and Pakistani Muslim communities in Britain, who make up nearly two thirds of the British Muslim population. Around 45% of these women have reported struggling with mental health [10, 11]. Young Pakistani and Bangladeshi mothers have also reported high levels of depression (37%) during the pandemic [12]. While this evidence is diverse and contributes to a broader understanding of mental health challenges amongst Muslim women, it is predominantly observational. However, some studies adopt qualitative approaches, offering meaningful insights from lived experience, though they limit the ability to draw firm conclusions about underlying causes.

Muslim women are known to experience high levels of Islamophobia in European contexts [13] and perceived discrimination in young British Muslim students in the UK is associated with increasing anxiety and depression, with social stigma remaining strong [14, 15]. The higher prevalence rates are likely to reflect a complex interplay of factors such as social disadvantage, discrimination, and acculturative stress [16, 17].

It is concerning that Muslims are under-referred to therapy services for mental health problems and show poor recovery rates compared to the general population, resulting in inequalities in mental health care and sub-optimal treatment for mental health problems [18, 19]. Muslim women face various barriers when seeking help for mental health. Within the South Asian community, cultural discourses such as ‘sharam’ (shame) and ‘izzat’ (honour) have a significant role. These concepts and the negative discourse of mental health in these communities have the potential to deter young South Asian women from seeking help as they fear bringing dishonour and shame to their families and communities in situations where poor mental health is seen as ‘defect’ [20]. In addition, lack of awareness about mental health, services available and what the help actually involves, family responsibilities, stigma around mental health and fear of being negatively judged by the community are additional factors which make it difficult for Muslim women to access mental health services [21, 22].

There is an unmet need for intervention that is greatest in underserved and ethnic minority communities [7]. This can be explained, in part, due to lack of tailored support to meet the needs of diverse groups with evidence that standard Cognitive Behavioural Therapy (CBT) based interventions are not as effective in promoting mental health and wellbeing in ethnic minority communities [23]. The Covid-19 pandemic highlighted the impact of health inequalities with increased mortality in ethnic minority communities and the importance of tailored support that considers social, cultural, and religious attitudes, beliefs and norms [13, 24]. Tailored support provided at a local level, such as health champions, demonstrated increased reach and engagement with formal services during the pandemic [25]. Religiously and culturally tailored interventions that encourage young Muslims to understand and discuss mental health, and improve help-seeking behaviours, overall mental health, and well-being, need to be developed and tested.

Evidence suggests that incorporating religious and cultural adaptations in tailored interventions for Muslim populations may improve mental health and well-being through ‘positive religious coping’, drawing on internalised spiritual beliefs to promote a deeper acceptance (this is due to mental health stigma and guilt), hope and resilience [18, 26–28]. Mental health services using such interventions in clinical practice can show promise as engaging religious beliefs can have a positive impact on psychological distress [27, 28]. Positive engagement with Muslim identity can also counter discriminatory practices, particularly for women who are disproportionately subjected to Islamophobic abuse, stereotypes, and violence [10, 29, 30]. Women from these communities are also subject to a ‘Muslim penalty’ with respect to education outcomes, economic activity, and unemployment [10, 31] which can contribute to mental ill health. Religious identity is core for many Muslim people and needs to be considered for person-centred care in this group [10, 26, 32]. Although religious and cultural identities can be closely intertwined with cultural expressions often influenced by religious themes, they are distinct dimensions of identity [33–35]. Concerningly, mental health service providers often lack an adequate understanding of religious and cultural sensitivities and cultural competence, leading to inappropriate or ineffective interventions [36]. In addition, cultural concepts relating to the causes of mental illness and social stigma amongst British Muslims combine with service mistrust to adversely impact service use [22, 37].

Muslim-led organisations offer mental health interventions delivered by therapists and counsellors trained in Islamic Psychology [38]. These take a holistic approach to religion within Muslim communities, which mainstream mental health provisions lack [38]. It is suggested that a collaborative approach involving faith communities, charities, and mainstream mental health services working together to improve religious and cultural competency amongst mental health practitioners would allow for knowledge exchange and increase access for Muslim communities who are often referred to as ‘hard-to-reach’ [38–40]. The Perinatal Mental Health support for Muslim communities, a collaborative project between Cambridgeshire, Peterborough, and South Lincolnshire (CPSL) Mind and The Lantern Initiative, is a success example of how different organisations can work together to support the needs of underserved Muslim communities [41].

Our research similarly follows a collaborative approach, bringing together academics who have expertise in research amongst Muslim populations and faith-communities more generally, practitioners who deliver culturally adapted therapy, and experts-by-experience members. Collaborating with Inspirited Minds (IM), a Muslim-led mental health charity, to deliver this faith-based and culturally adapted therapy programme—IM-Adapted—will support our aim of establishing how to engage participation from within Muslim communities. Despite the growing evidence, impacts of mental health on Muslim communities and minority ethnic groups are underrepresented in research [42–44]. Some of the barriers to participating in research for minority groups include language, not knowing about research, stigma, trust, location, cultural awareness and competence, culturally matched researchers, and understanding the consent process [43, 45]. This feasibility study will be key for establishing whether our current strategies for recruitment and retention of participants have worked and whether strategies to optimise participation need to be adopted for a full randomised controlled trial (RCT) to take place.

Our research will address key public health challenges, including how best to engage with communities who do not access mental health services, delivering community/faith-based interventions in non-NHS settings to support young women, training practitioners in this novel approach, and using appropriate evaluation frameworks to capture process and outcomes [46, 47]. It will inform a pathway to testing the effectiveness and cost-effectiveness of the IM-Adapted intervention for young Muslim women, increasing representation of marginalised groups in research.

### Objectives

To establish the feasibility of conducting a full RCT of a faith-based culturally adapted intervention to promote and improve mental health and well-being of young Muslim women in community settings. It will determine whether it is possible to recruit and retain young Muslim women to the trial, develop and deliver the intervention as planned, evaluate the training and delivery of the intervention, explore the feasibility of collecting data, and will provide data to inform the design of the main trial.

### Trial design

A two-arm cluster randomised controlled feasibility trial delivered in groups in two locations (Birmingham and London). Clusters will each comprise 8–10 young Muslim women, with three groups at each site. This trial incorporates embedded mixed methods process evaluation.

## Methods: participants, interventions, and outcomes

### Study setting

The IM-Adapted feasibility trial will take place in Birmingham and London. The intervention will be delivered by trained mental health professionals, employed by the Inspirited Minds charity, in local mosques or community centres.

### Eligibility criteria

Individuals are eligible for inclusion if they are a Muslim woman aged 18 to 24 years, self-diagnosed or referred from another source with low mood or mild depression and scoring between 5 and 14 on the Patient Health Questionnaire 9 scale (PHQ-9) [48], who are willing to take part in the study, consent to participate, and are able to read and understand English.

Individuals will be excluded from the study if they do not meet the above eligibility criteria and are considered unsuitable based on the professional opinion of the IM’s mental health professionals eligibility screening and risk assessment (e.g. presenting significant risk) or their current treatment or co-morbid conditions present contraindications to engaging in the trial (e.g. severe chronic pain).

### Interventions

Participants will be randomised in groups of approximately 10, as soon as sufficient numbers have accrued at a site. Groups will be allocated to receive either the Inspirited Minds Adapted (IM-Adapted) programme (intervention arm) or the usual psychoeducation support group (control arm). The sessions will be run by a trained therapist supported by a mental health support worker (MHSW) and will be delivered once a week for 6 weeks, lasting approximately 90 min in a mosque or community centre.

#### Inspirited Minds adapted (IM-Adapted) intervention arm

For the purposes of this intervention, we define religion as “an organised system of beliefs, practices, and symbols designed to facilitate closeness to the sacred or transcendent (God, higher power, or ultimate truth/reality), and to foster an understanding of one’s relationship and responsibility to others in living together in a community” [49]. The IM-Adapted programme is a faith-based culturally adapted peer support group intervention aimed at promoting and improving mental health and well-being and tailored to the specific needs of Muslim communities. It was co-developed with Muslim service users and therapists. The IM-Adapted intervention (see Table 1 for key characteristics) is CBT-based and adapted with Islamic faith messages using cognitive and behavioural underpinnings including insights from GM’s (co-applicant) therapeutic approach with Muslim populations [18, 28] and CBT models used in cultural adaptations [50–52]. The intervention also uses a place-based community approach [53] by delivering in a mosque or community centre. It is delivered by Muslim women who are experienced practitioners and have previously been trained in the use of CBT by the NHS and co-facilitated with mental health support workers. Inspirited Minds staff delivering the IM-Adapted programme will receive additional training in the delivery of IM-Adapted via a training manual coupled with online workshop sessions.

**Table 1:** Comparison of key characteristics of intervention and control arm group sessions

IM Adapted	Control group
Group run by Muslim women with standard NHS CBT training who have additionally received specialised training in the delivery of the IM-Adapted intervention	Group run by Psychological Wellbeing Practitioners who are Muslim women with standard NHS CBT training
Starting the treatment with the Dua (Islamic prayer)	Normal introductions to the session
CBT Components including the 5 areas model [56]	CBT components including the 5 areas model [56]
Supportive space to have open conversations, using questions around culture and religiosity to prompt discussion and hearing from others	Supportive space to have conversations without religious and cultural prompting
Islamic content and stories related to depression from the Quran and stories of the time of the prophet, having discussions around this	Not included
Homework setting	Homework setting
Journaling every week allowing time to reflect on cultural and religious learning from the sessions with prompted questions	Non-culturally or religiously informed journaling activity
Ending with cultural and religious reflections and key learning	Ending with general reflections and key learning
Ending with a Dua (Islamic prayer) for hope	Not included
Being provided with IM booklets around mental health, which is faith based	Booklet and content from the slides

The intervention recognises the broader cultural identities of young Muslim women and addresses issues of stigma, social structures, and religious explanations for depression. This involves creating positive messages from Islamic teachings that relate to positive religious coping, behavioural components linked to behaviour change theories, activity scheduling to address negative thoughts and improve mood and impact on behaviour, use of positive logs, journalling, goal setting, modelling positive behaviours, and the use of peer support to tackle loneliness and offer initial steps to improve low mood, address barriers, and improve access to support. This results in reduced stigma and allows individuals to feel more confident to seek support. It focuses on addressing negative beliefs and positive and negative coping through religious messages, thereby increasing positive beliefs about mental health [54, 55].

Themes addressed by IM-Adapted are the following:

- Culture and related issues including a lack of communication and understanding around mental health, a taboo subject, with guilt and beliefs of not being a good Muslim if you are struggling with your mental health.
- Capacity and coping strategies including limited adapted treatments to suit more Islamic ways of working and using faith as a way of coping and sometimes leading to use of recreational drugs to cope due to needing to mask symptoms.
- Cognition and beliefs, including rigid beliefs about mental health and assumptions that if people pray more, they will feel better.
- Isolation and stigma due to limited access to specialised treatments and not openly communicating with others and hence feeling like they are the only ones struggling with mental health.

#### Psychoeducation support group control arm

The comparison group is a low-intensity CBT psychoeducation group with no religious or cultural adaptation (see Table 1) delivered by qualified (Mental health professionals who have a core profession in psychology, for example, Psychological Wellbeing Practitioners, Counsellor, CBT Therapist) female Muslim mental health professionals from IM. As with the IM-Adapted intervention arm, control participants will receive six weekly sessions (90 min per session) in groups of approximately 10 women. The sessions will include components of CBT in a group format for low mood, including behavioural activation, activity scheduling, thought challenging, and understanding their pattern using a 5 areas diagram.

### Outcomes

The primary feasibility outcomes to determine progression to a future definitive trial are as follows:

- Number of participants referred, excluded, and recruited and reasons for not participating
- Retention rates at follow-up
- Participant engagement and adherence (sessions attended)
- Feasibility, acceptability, barriers and facilitators to interventions (process evaluation)
- Data quality and follow up completion rates, including health economic data
- Training and delivery model (adherence, engagement, confidence)
- Adverse event rate
- The existence of early evidence that the intervention is not clearly inferior to usual care
- Feasibility of required sample size

We will measure a range of secondary outcomes with a view to making a decision about which to include in a future definitive trial. The secondary outcome measures (as potential primary outcomes for a definitive trial) are the following:

- Distress, well-being, anxiety, overall health-related quality of life, loneliness, resource use and religious coping.

### Participant timeline

Table 2 shows the SPIRIT (Standard Protocol Items: Recommendations for Intervention Trials) [57] table detailing study processes.

**Table 2:** SPIRIT (Standard Protocol Items: Recommendations for Intervention Trials)

	Study period						
	Screening	Randomisation	Post-randomisation				
Timepoint	Pre-T0	Pre-T0	T0	T0–T1	T1 + 6 weeks	T2 + 24 weeks	
**Enrolment:**							
Eligibility screen	X						
Referral to research team	X						
Informed consent	X						
Randomisation		X					
**Interventions:**				X			
**Assessments:**							
PHQ-9	X		X		X	X	
Demographics information	X						
GAD-7			X		X	X	
WEMWBS			X		X	X	
R-COPE			X		X	X	
EQ-5D-5L			X		X	X	
CSRI			X			X	
Measure of loneliness			X		X	X	
Adherence				X			
Focus groups							X
Adverse event monitoring				X	X	X	

*Note*: *T0* = baseline, *T1* = first follow-up, *T2* = second follow-up, *PHQ-9* = Patient Health Questionnaire, *GAD-7* = Generalized Anxiety Disorder, *WEMWBS* = The Warwick-Edinburgh Mental Wellbeing Scale, *R-COPE* = Brief Religious Coping Measure, *CSRI* = Client Service Receipt Inventory

### Sample size

A sample size of 60 eligible young Muslim women from Birmingham and London (30 per site and 30 per arm) has been selected to enable 24 participants per arm at the final follow-up point, allowing for an estimated loss to follow-up of up to 20% (co-applicant GM’s study unpublished [58, 59]. This sample size allows for at least one IM-Adapted intervention group to be completed at each of the two study sites, giving six groups in total (3 intervention; 3 control). Each group is required to have 10 participants to ensure that at least 8 participants are present at any given session (allowing for 20% no show/drop out). As this is a feasibility study, the sample size is not powered to evaluate the efficacy of the intervention. A sample size of 60 participants will allow estimation of the completion rate to within a pre-specified level of precision of ± 10% assuming it is 90%, or ± 13% if it is 80%. We are confident that these are appropriate estimates and represent values that would allow progression to a full trial. The sample size is also in line with those for feasibility studies audited in the UK [60].

### Recruitment

Young women will be informed about the study through mosques and community groups supporting recruitment for the study and through IM’s usual referral processes, study website, and social media. Individuals will be directed to complete the online self-referral form held on the IM website.

Study staff at IM will review incoming referrals to their services, via their usual online form, to identify those who meet the initial eligibility criteria check (i.e. are the right age, gender and have reported low mood/depression scoring 5–14 on the PHQ-9). Those meeting the initial eligibility check will be contacted to introduce the study and set up a consultation via telephone or video call. This includes a standard risk assessment undertaken by IM staff to finalise eligibility for participation in the study.

Those who are ineligible based on risk or other eligibility criteria or do not wish to participate in the study will be offered the usual support provided by IM as appropriate. These individuals will be offered the option to give reasons for why they do not wish to participate in the study, but this question will be optional to avoid apparent coercion.

Those who meet all eligibility criteria for the study and would like to take part will be sent the link to the online (via REDCap) informed consent form.

## Methods: assignment of interventions

### Allocation

Participants will be recruited and placed sequentially into a group at each site; once the group at the site has reached 10 participants, the group will be allocated. The allocated treatment for a group will be automatically generated via the study database. Stratification will be by study site (Birmingham or London) with a block size of 2. The groups will be allocated by a process embedded in the web-based data management system REDCap [61, 62] managed by the University of Hertfordshire Clinical Trials Support Network (UHCTSN) ensuring allocation concealment. When a group is randomised, an email will be sent to the principal investigator (PI) and the Trial Manager, who will communicate with the Therapist and MHSW to set up the appropriate group sessions.

Due to the nature of the intervention, allocation blinding is not possible. Where possible, both supporting staff will be blinded during data collection and statistical analyses will be blinded. 

## Methods: data collection, management, and analysis

### Data collection methods

Screening data will be collected from the IM database resulting from self-referrals. Participant data will only be entered directly into the study database from the time of informed consent. Data collection, data entry, and any queries will be managed in line with the UHCTSN and trial-specific data management processes.

#### Baseline and follow-up

Participants will be followed up at 6 weeks and 24 weeks. They will receive a link by email to complete outcome measures as online questionnaires on their own devices. An option of telephone data collection will be offered to those participants who would prefer it. To maximise engagement, reminders will be sent via email to those who have not completed the questionnaires and followed up by phone call when necessary; participants will be verbally reminded during the first and last session of when to expect the link to complete measures, and an option of telephone data collection will be offered to those participants who would prefer it.

Attendance, adherence and adverse event data will also be entered into the study database by IM staff and reviewed by the Trial Manager and Chief Investigator (CI) where required. Participants are able to withdraw at any point after enrolling in the study; however, any data collected up to that point will be kept as detailed in the participant information sheet.

#### Outcome measures

Psychological measures.

- Patient Health Questionnaire-9 (PHQ-9) [48]: a 9-item measure which assesses self-reported severity of depression.
- Generalized Anxiety Disorder (GAD-7) [63]: a 7-item measure which assesses self-report anxiety.
- The Warwick-Edinburgh Mental Wellbeing Scale (WEMWBS) [64]: a 14-item measure with 5 response categories which enables the measuring of mental wellbeing.
- Brief Religious Coping Measure (R-COPE) [54] adapted for a previous trial [65] to include additional questions from Kahn and Watson [55].
- Direct Measure of Loneliness [66].

Additional measures for use in the economic evaluation:

- EQ-5D-5L: [67] a five-item measure to assess Health-Related Quality of Life (HRQoL) [68].
- EuroQol Visual Analogue System (EQ-VAS); a visual analogue scale to assess health status on a scale of zero to 100 to measure health status [67].
- Modified Client Service Receipt Inventory (CSRI) [68]: a widely used instrument to collect information on services and support used by study participants.

#### Progression criteria

The following criteria have been developed to determine whether funding for a definitive trial will be sought, including items relating to recruitment, retention, adherence, and completion. This progression criteria follow a set of stop–go criteria (based on traffic light colours for the measures, where appropriate). Where traffic lights are used, green = progression (subject to a successful new funding application), amber = modifications and improvements required before progressing, and red = no progression to a future trial. For the non-traffic light measures listed below, a decision will be made based on the information available.

- Completion of key outcome measures (% of mental health and well-being measures completed):
  - ◦ Green: > = 80%
  - ◦ Amber: 51–79%
  - ◦ Red < = 50%.
- Participant engagement and attendance (% of planned sessions attended):
  - ◦ Green: > = 67%
  - ◦ Amber: 51 to 66%
  - ◦ Red: < = 50%
- Successful collection of resource use data (% of participants providing resource use data):
  - ◦ Green: > = 80%
  - ◦ Amber: 70–79%
  - ◦ Red: < 70%
- Adequate acceptability of interventions to young Muslim women.
- Professionals and support workers are willing to deliver sessions and encourage participant engagement.
- Sufficient referrals (with sufficiency based on the sample size required for a definitive trial, we will calculate from this feasibility study) received from various organisations for young people to participate in the trial.
- Recruitment is such that > = 10% of eligible screened participants are recruited.
- Appropriate outcomes measures are identified, including one potential primary outcome (> 75% of minimum important difference).
- Based on the estimated sample size, a necessary number of additional sites is identified for a definitive trial. No indication that the intervention is significantly less effective than usual care.
- No indication that the intervention has significantly increased the risk of adverse events compared to the control condition.

#### Safety

Adverse events will be recorded throughout the course of the trial.

#### Process evaluation

Our embedded mixed methods process evaluation will aim to identify key issues and potential solutions to inform the design of a full-scale trial [47]. In particular, it will explore the effectiveness of recruitment pathways, the acceptability of the study design and intervention, and fidelity to the intervention protocol and training.

Mixed method approaches will include the following:

- Intervention logs completed by the therapists, which will include the number of young people attending, absences/partial attendance, and if planned activities went ahead.
- Observations of 10–15% of intervention sessions using an observation log to examine implementation and fidelity to the training and manual.
- Focus groups with therapists and support workers involved in intervention delivery (all staff will be invited, and we will carry out 1–2 focus groups per site). This will explore experiences of intervention training, delivery, and study processes.
- One focus group with the IM staff involved in delivery to gather experiences of running the intervention and any modifications that may be required or anticipated before progressing to a definitive trial.
- Focus group/interviews with referral partners to gather their experiences of referring their clients or service users to the intervention.
- Interviews or focus groups with approximately 24 women in total, aiming for 12 from each arm and equal numbers from both sites. These will explore their views on the acceptability of the intervention and study design and procedures, along with barriers and facilitators for engagement. They will be facilitated by someone of Islamic faith. Where possible, purposive sampling will be used to ensure diversity of experiences and perspectives.

The process evaluation draws on the Theoretical Framework of Acceptability (TFA; [69]) and will primarily explore retrospective acceptability via interviews and focus groups. Our intervention observations and intervention logs will also provide insight into concurrent acceptability, for example relating to opportunity costs (e.g. if participants are late to intervention sessions because of other commitments) and burden (e.g. issues with completion of homework).

Semi-structured topic guides will be developed for all focus groups and interviews. These will be informed by the TFA [69], and we will liaise with the Young Women’s Advisory Group (YWAG) as appropriate. For convenience, we anticipate interviews and focus groups will all be undertaken online but will be in accordance with participant preference.

### Data management

The database assigns participants with a unique participant identifier (PID) and all data will be recorded against this identifier. The database will be controlled, administered, and managed by the UHCTSN Data Management team. To maintain data quality, an audit trail will be maintained, allowing data queries to be raised, and missing data will be monitored. Further information can be found within the Data Management and Access Plan attached to our funding award (NIHR156425).

#### Statistical methods

Data analysis will be largely descriptive, reflecting the fact that this is a feasibility study where the aim is not to test the efficacy of the intervention but to assess the feasibility of conducting a larger scale trial. Based on our previous work [70], the referral, recruitment, and retention will be evaluated using standard reporting following the CONSORT [71, 72] criteria, reporting the proportions (and confidence intervals) of young people reaching each stage of the study, by referral source and study arm. Adherence will be assessed through the proportions of sessions attended and the extent to which young women continue to apply strategies learnt to improve mental health (survey data at 24 weeks). Feasibility of collecting outcome data will be evaluated by estimating the proportions of missing data in each of the outcomes assessed. The descriptive statistics of the outcome measures will be given by study arm, but no formal hypothesis testing will be undertaken. For the outcome measures, we will use the intention-to-treat population, i.e. individuals will be included if data are available in the study arm to which the group they are in was allocated regardless of attendance or compliance. As this is a feasibility study, no imputation of missing data will be carried out. To monitor safety, the number of adverse events (for example, a mental health episode triggered by the content of a session) will be reported by study arm along with the number of participants experiencing one or more events for individuals who attend at least one group session. Recruitment to the study will be monitored through the recruitment logs and reported using the standard CONSORT criteria for feasibility studies.

#### Process evaluation

Qualitative focus group and interview data will be audio recorded and transcribed. If conducted online, Zoom transcription will be enabled and quality checked and corrected in conjunction with the audio recording. The data will be analysed using reflexive thematic analysis [73] on NVivo software. We will use both deductive coding (based on the Theoretical Framework of Acceptability) and inductive coding (to centre the experiences and perspectives of participants) to develop themes. This will provide insight into recruitment, engagement, and delivering and receiving the intervention. Data from the intervention logs and intervention observations will be compared against the intervention manual, and findings will be tabulated and summarised narratively to inform our understanding of fidelity to the training and how the intervention was delivered and received. Triangulation of the analysis of the focus groups and interviews, intervention observations, and intervention logs will be used to describe delivery of both arms and examine implementation fidelity and explore factors affecting engagement across study processes. This will inform the feasibility assessment of the study and refine the design and intervention for a future trial.

#### Economic evaluation

The methods required to evaluate cost-effectiveness of a future trial will be tested in this feasibility study. We will measure resources required to provide the IM-Adapted intervention and the comparator of psychoeducation group support in the two groups. Resources will include staff time, equipment and consumables, accommodation, and staff training. In addition to these resources, an effective intervention may affect the use of health and social care services, as well as costs borne by the young women and their families. These will be recorded by means of a resource use questionnaire (modified client service receipt inventory (CSRI)) at baseline and 24 weeks [68], which, at both baseline and follow-up, will ask about the previous 24-week period. This will also record important non-health-related resource implications, including employment, education, or training status and time off work or usual activities. The modified CSRI cover aspects related to non-health-related resource use, including employment status, time unemployed, time off work or usual activities. It will also cover health-related resource use of inpatient and secondary care services, community and primary care services, and use of medicines. Resource use categories were discussed with other members of the research team to ensure that the most relevant categories of relevant service use were covered. Part of this feasibility study will include an assessment of this instrument, including completeness and ease of use. Any resources identified by the CSRI will be costed using appropriate local and national cost data.

The main outcome measure for the economic evaluation in a future main study would be the Quality Adjusted Life Year (QALY). In the feasibility study we will estimate QALYs using the EQ-5D-5L, collected at baseline, 6 and 24 weeks [68], and scored using a published scoring algorithm [74]. In a future full economic analysis, the EQ-5D-5L would be used to generate QALYs using ‘area under the curve’. The EQ-5D-5L will be compared descriptively with other outcome measures used in the feasibility study. It is not intended to estimate cost-effectiveness from the feasibility study as the study will not be powered to demonstrate this. Rather, the aim is to trial the proposed health economics measures, ascertain their completeness and acceptability to respondents, and identify likely main drivers of costs.

## Methods: monitoring

### Data monitoring

Data monitoring and monitoring of participant safety will be allocated as a standing agenda item for the Trial Steering Committee (TSC). Where data monitoring indicates that serious harm might be occurring in more than two cases over the course of the study, a subgroup of the TSC will be asked to review the data and make recommendations relating to likely future risk to the study participants and the need for further independent scrutiny of the data.

### Harms

To monitor safety during the study, any adverse event (AE) occurring during the participation of the study will be reported in accordance with the study protocol and IM policies. We will monitor for any potential harm and unanticipated outcomes during the intervention and the 6-month follow-up period. Should an AE occur, the member of the study team who first becomes aware of the AE must record it on the database and assess whether the event is considered serious. All serious adverse events (SAEs) will be communicated to the PI and Trial Manager (or delegated person in their absence) within one working day using the provided SAE form on the database. The PI and Trial Manager will review the SAE form and disseminate it to the CI and the Trial Management Group (TMG) within 72 h of being informed, where appropriate. The TSC and Research Ethics Committee (REC) will be informed by the Trial Manager of all SAEs periodically unless the Chief Investigator (CI) escalates the SAE or deems it necessary.

### Auditing

Quality assurance activities will be conducted by the sponsor and UHCTSN. This will include following the UHCTSN quality management system and review of key documents. Central and site monitoring will be conducted during the course of the study. An onsite monitoring visit at each site will be conducted at least once during the study, and central monitoring of the database will occur throughout the study.

## Public involvement

Public involvement in the study is co-led by a member of the research team and a public co-applicant with relevant lived experience. Participative approaches are embedded throughout the project, including in the development of the proposal. This included a workshop in November 2022, attended by six women with relevant lived experience and supported by funding from the NIHR Research Design Service. This identified the need for young people’s involvement to be inclusive and flexible and to address issues about stigma, cultural sensitivities, and religious guilt. Fifteen young Muslim women aged 16 to 30, with lived experience of using mental health services, were identified through the authors’ networks and invited to attend an online workshop in November 2023. Ten attended and contributed to the development of plans for inclusive involvement in the study.

In Spring 2024, an invitation was sent out via the authors’ networks for UK-based young Muslim women, aged 18–30 and with lived experience of using mental health services and/or affected by mental health issues, to join a public advisory group. Twelve expressions of interest were received and the first meeting of the Young Women’s Advisory Group (YWAG) took place in April 2024. The group will meet every 2–3 months, depending on group members’ availability and the stage of the project. The meetings will mainly take place online, with the option for in-person or hybrid meetings/social events if this works for group members.

Training and support will be provided as needed. Members of the YWAG will also have opportunities for involvement outside of meetings (e.g. reviewing documents, attending events), as well as having two YWAG places on the TSC and being supported to have an active role in the process evaluation. Members of the YWAG will receive gift vouchers as a thank you for their involvement.

Public involvement in the study is overseen by a working group led by the public involvement lead and the public co-applicant, which includes the CI and trial manager. The group meets monthly to collectively ensure that plans for involvement are embedded throughout the project, are meaningful and effective, and reflect cultural sensitivities and lived experience, reporting regularly to the TMG and TSC. The public co-applicant will provide a ‘lived experience’ perspective throughout the project, working with the public involvement lead to plan and co-facilitate public involvement, and supporting and advising the research team on working in culturally sensitive and inclusive ways. We will also draw on support from the UH public involvement team and the NIHR Applied Research Collaboration East of England’s public involvement workstream where required.

## Ethics and dissemination

### Research ethics approval

Ethical approval was received from the Health, Science, Engineering and Technology Ethics Committee with Delegated Authority (ECDA) (Protocol Number: HSK/SF/UH/05701). Informed consent to participate will be obtained from all participants.

### Protocol amendments

To communicate protocol changes, we will promptly notify all stakeholders, including trial investigators, participants, and the approving ethics committee, through formal emails and updated documentation. Major modifications will be shared on the study’s website to ensure clarity and transparency. Additionally, regular updates will be provided during scheduled meetings with the research team, TSC, and public advisory group stakeholder forums to maintain consistent messaging.

### Consent

Eligible young women will be asked to complete the online informed consent form. This form can be accessed by a link which will take them to the online form via the REDCap database held by the University of Hertfordshire. Participants will receive a copy of the signed informed consent form via email.

Consenting eligible women will then be recruited to the study and randomised either to the IM-Adapted intervention or the comparison group.

### Confidentiality

Data collection will include sensitive details such as demographic information, mental health assessments, and qualitative feedback, gathered through secure methods like encrypted digital forms and password-protected databases. Access to this information will be restricted to authorised personnel involved in the trial, who will be trained in confidentiality protocols. During the trial, data will be anonymised using unique participant codes to minimise risks. Sharing of data will be limited to essential purposes such as analysis and monitoring, and this will occur in a de-identified format to uphold privacy. Post-trial, all personal information will be securely archived or disposed of in accordance with data protection regulations, ensuring participants’ details remain confidential beyond the study period.

### Access to data

While the study is ongoing, and prior to publication of the final dataset, access to the final trial dataset will be restricted to authorised members of the research team, including the lead investigator and key co-investigators involved in the data analysis. The data manager, responsible for maintaining the dataset, will ensure secure storage and controlled access. Any sharing of the dataset with external collaborators or stakeholders will require explicit permission from the lead investigator and will be governed by the research agreement and data sharing protocols.

After the final study data is published in a peer-reviewed article (normally within 18 months of the study end date), an anonymised dataset will be made publicly available on the University of Hertfordshire Research Archive. Interview and focus group data will not be made publicly accessible due to the difficulty of making this data anonymous. Access to the data will be granted provided that the article reporting the final data is quoted in any reports.

All team members and collaborators must adhere to the contractual agreements and ethical guidelines that restrict the use of the dataset solely to the agreed research purposes. There are no contractual agreements that limit investigator access to the dataset, ensuring transparency and integrity of the research findings.

### Ancillary and post-trial care

Following participation in the study, those who took part will still be eligible for support from IM, and signposting will be available.

### Dissemination policy

Plans for investigators and sponsors to communicate trial results to participants, healthcare professionals, the public, and other relevant groups (e.g. via publication, reporting in results databases, or other data sharing arrangements), including any publication restrictions.

The findings will be published in peer-reviewed journals and presented at relevant conferences aimed at public health and mental health professionals. Additionally, results will be shared on the project’s website and through social media channels to engage and inform a wider audience, including participants and stakeholders. Public and stakeholder involvement will be central to the dissemination strategy, which will include reporting outcomes to networks, including mental health services, voluntary organisations, and community groups. The results will also be reported in relevant results databases, adhering to data sharing arrangements and ethical guidelines. Any restrictions on publication will align with standard practices to protect the integrity of the trial, ensuring that findings are disseminated transparently without undue delay.

### Trial status

Currently recruiting.

## Data Availability

Not applicable. No datasets are included in this study protocol.

## Abbreviations

AE: Adverse event
CI: Chief investigator
CTSN: Clinical Trials Support Network
IM: Inspirited Minds
IM-Adapted: Inspirited Minds (IM)-Adapted
ISRCTN: International Standard Randomised Controlled Trial Number
MHSW: Mental Health Support Worker
PI: Principal Investigator
PID: Participant Identifier
QALY: Quality adjusted life year
RCT: Randomised control trial
REC: Research Ethics Committee
SAE: Serious adverse event
TMG: Trial Management Group
TSC: Trial Steering Committee
YWAG: Young Women’s Advisory Group
